# Molecular epidemiology of Enteroviruses and Rhinoviruses in patients with acute respiratory infections in Yaounde, Cameroon

**DOI:** 10.1111/irv.12851

**Published:** 2021-03-10

**Authors:** Sebastien Kenmoe, Serge Alain Sadeuh‐Mba, Marie‐Astrid Vernet, Véronique Penlap Beng, Astrid Vabret, Richard Njouom

**Affiliations:** ^1^ Virology Department Centre Pasteur du Cameroun Yaounde Cameroon; ^2^ Département de Biochimie Université de Yaoundé 1 Yaounde Cameroun; ^3^ Normandie Université Caen France; ^4^ UNICAEN UNIROUEN GRAM Caen France; ^5^ Department of Virology University Hospital of Caen Caen France

**Keywords:** Cameroon, Enterovirus, molecular characterization, respiratory infections, Rhinovirus

## Abstract

**Background:**

Acute respiratory infections (ARI) are associated with a huge morbidity and mortality worldwide. Rhinoviruses (RVs) and Enteroviruses (EVs) are recognized as leading causes of ARI.

**Objectives:**

The present study describes the molecular epidemiology of RVs and EVs in Cameroon over a 3‐year surveillance period.

**Methods:**

From September 2011 to October 2014, nasopharyngeal swabs were collected from patients with influenza‐like illness (ILI) and severe acute respiratory infections (SARI). Two sub‐genomic regions of the EVs and RVs were targeted for molecular characterization. These included the most conserved 5′‐untranslated region (5′UTR) and the viral protein 4/viral protein 2 transition region (VP4/VP2).

**Results:**

A total of 974 samples were collected. Children ≤5 years accounted for 85.7% (835/974) of all participants. Among them, 160 (16.4%) were positive for RVs and/or EVs. RVs and/or EVs were significantly more identified in ILI compared to SARI patients (*P* = .015). Both viruses co‐circulated all year long with a marked increase of occurrence during rainy and cold season. All RV species were found to circulate in Cameroon, with 6, 10 and 6 virus types belonging to the RV‐A, RV‐B and RV‐C, respectively. EV species identified comprised EV‐A (1 Coxsackie virus A5), EV‐B (1 Coxsackie virus A9 and 2 Coxsackie virus B1) and EV‐C (1 EV‐C117).

**Conclusions:**

This study indicates a strong year‐round occurrence of EV and RV associated respiratory infections in Cameroon. Molecular characterization identified a wide variety of RVs and EVs in patients with ARI in Cameroon.

## 1 INTRODUCTION

Acute respiratory infections (ARI) constitute a major cause of morbidity and mortality worldwide. Although global mortality due to lower respiratory tract infection (LRTI) and upper respiratory tract infection (URTI) underwent a reduction of 21.1% and 42.1%, respectively, from 2007 to 2017, these infections still caused more than 2.5 million deaths in 2017.^1^ The burden of LRTI was worsened even more with the emergence of COVID‐19 in late 2019 which has already caused more than 2.4 million deaths worldwide in less than a year.^2^ Respiratory viruses are also responsible for huge economic losses due to the use of health care resources and absenteeism at work and school.^3^, ^4^, ^5^ Respiratory viruses, including Rhinoviruses (RVs) and Enteroviruses (EVs), are the most common cause of ARI. Rhinoviruses are the main cause of URTI worldwide with common cold being their main clinical presentation.^6^, ^7^ With the development of molecular detection assays, RVs have recently been shown to be also involved in a significant proportion of more severe LRTIs such as wheezing, bronchiolitis, asthma exacerbation and chronic obstructive pulmonary disease.^8^, ^9^, ^10^ Rhinoviruses have also been reported in apparently healthy individuals, possibly due to the prolonged viral shedding period.^11^, ^12^ Enteroviruses, on the other hand, have a broader tropism and can cause a wide range of human infections including acute respiratory infections, meningitis, encephalitis, gastroenteritis, acute flaccid paralysis or conjunctivitis.^13^, ^14^, ^15^ A new strain of EV‐D68 re‐emerged, circulated worldwide and caused particularly severe acute respiratory infections (SARI) and acute flaccid myelitis.^16^

Enteroviruses and RVs belong to the *Picornaviridae* family and the *Enterovirus* genus. According to the International Committee on Taxonomy of Viruses (ICTV), the *Enterovirus* genus comprises 15 species EV‐A to L and RV‐A to C.^17^ Based on the sequence diversity of the structural protein coding genes, individual viruses of the EV species are further assigned into virus types. Rhinoviruses consist of more than a hundred virus types. Virus type assignments based on the nucleotide sequence of the VP4/VP2 region correlate with the type assignment based on the VP1 region of EVs and RVs.^18^, ^19^

All RV and EV species have a global distribution and a year‐round circulation pattern with occasional peaks.^20^ In West Africa,^21^, ^22^, ^23^ Southern Africa,^24^, ^25^ Northern Africa,^26^, ^27^ Eastern Africa^28^, ^29^, ^30^ and Central Africa,^31^, ^32^, ^33^ multiple studies reported the circulation of EVs and RVs in patients with mild and severe respiratory infections. However, data on genetic diversity and circulating types remained limited. Most of the studies have been conducted outside of Central Africa and have focussed on strains of EV‐64.^24^, ^34^, ^35^, ^36^, ^37^, ^38^, ^39^, ^40^ In Cameroon, RVs and EVs have been shown to be among the most commonly viruses detected in patients with LRTI and URTI,^31^, ^41^ but the virus types involved remain unknown. In the present study, we report the occurrence and phylogenetic relatedness of EVs and RVs detected in samples from patients of all ages suffering from ARI in Cameroon based on the sequences of the VP4/VP2 genomic region.

## 2 METHODS

### 2.1 Study design

In this cross‐sectional study, we conducted a prospective recruitment of patients of all ages at Centre Hospitalier d'Essos in Yaounde, Cameroon. We included participants enrolled from September 2011 to October 2014. Ethical approval was obtained from the National Ethics Committee of Research for Human Health (N° 121/CNE/SE/2011) and an administrative authorization from the Ministry of Public Health of Cameroon. Patients were informed of the study objectives, constraints, risks and benefits of participating in the study and those who provided their informed consent were enrolled. We included outpatients and inpatients with acute respiratory infections. A case of ARI was considered as any patient with a fever <5 days in addition to a cough and/or sore throat. Participants who did not provide their consent were not included.

### 2.2 Laboratory analysis

We collected a nasopharyngeal swab from all participants in a tube containing 1 mL of viral transport medium. Sample was shipped to the laboratory in reverse cold chain and stored at minus 80°C until the time of analyses. Viral RNAs were extracted and purified using the QIAamp Viral RNA Mini kit (Qiagen) according to manufacturer's specifications. A preliminary detection of RVs and/or EVs was carried out by real‐time RT‐PCR using primers and probes from the commercial Respiratory Multi Well System Rhino&EV/Cc r‐gene (BioMérieux) according to the manufacturer's intructions. Most of these RV and/or EV positive samples had already been the subject of several other studies, and the remaining volumes were very limited.^41^, ^42^, ^43^, ^44^, ^45^ Overall, 43 EV and/or RV positive samples have swab volumes were sufficient to allow RV and/or EV amplification and sequencing and were therefore considered in this study. We amplified the VP4/VP2 region of 43 RV and/or EV positive samples using the RT‐PCR technique previously described by Linsuwanon et al,^46^ in 2009. This RT‐PCR technique generates an amplicon of 540 nucleotides encompassing a fragment of the 5′‐UTR gene, the VP4 gene and a fragment of the VP2 gene. RNAs were initially used as a template to synthesize complementary DNA using random primers and the SuperScript™ III First‐Strand Synthesis System kit (Invitrogen). Resulting complementary DNAs were subsequently amplified in a first round PCR using the AmpliTaq DNA polymerase kit (Invitrogen) using primers F484 (5′‐CGGCCCCTGAATGYGGCTAA‐3′) and R1126 (5′‐ATCHGGHARYTTCCAMCACCA‐3′). The products of this first PCR were finally amplified in a second round PCR using the FastStart™ Taq DNA Polymerase kit (Roche) using primers F587 (5′‐CTACTTTGGGTGTCCGTGTTTC‐3′) and R1126 (5′‐ATCHGGHARYTTCCAMCACCA‐3′). Amplicons from the second PCR by gel green stained 2% agarose gel electrophoresis. Second round PCR amplicons were sequenced using the BigDye Terminator 1.1^®^ kit (Thermo Fisher Scientific) according to the manufacturer's recommendations.

### 2.3 Sequence analysis

Sequences were assembled and edited using EDITSEQ of the software Seqman™ II Lasergene (DNA). RV and EV reference sequences were retrieved from the GenBank database. Multiple sequence alignments were carried out using the CLUSTAL W method^47^ in the MEGA software version 6.^48^ Originally, we used the neighbour‐joining method with a 1000‐bootstrap replicates to generate an extended phylogenetic tree with all known EV types in one hand and all known RV types in the other hand (Figures S1 and S2). Then, the EV and RV sequence data sets were downsized by omitting most branches containing no studied sequences. Reduced data set was used for maximum likelihood phylogenetic analyses using the best fit evolution model which was General Time Reversible with a Gamma distribution and assuming that a certain fraction of sites was invariable on an evolutionary level (GTR + G + I). The reliability of the tree nodes was evaluated by 1000 bootstrap resampling. Nucleotide sequences obtained in the present study were submitted to GenBank under the registration numbers MN508757 to MN508783.

### 2.4 Statistical analysis

We collected clinical and sociodemographic data from participants using a standardized form. We analysed the data using R software version 3.6.0. We described the categorical variables in number and percentage and the continuous variables in mean and standard deviation. We used the chi‐square test to compare categorical variables and linear regression for continuous variables, and *P* values < .05 were considered statistically significant.

## 3 RESULTS

### 3.1 Sociodemographic and clinical characteristics of the patients enrolled

From September 2011 to October 2014, we recruited 974 participants in this study. The age of the participants ranged from 1 month to 59 years with a mean of 3.3 ± 5.9 years. The proportions of the age groups represented varied inversely depending on the age of the participants: <2 years, 597 (61.3%); 2‐5 years, 238 (24.4%); 5‐15 years, 108 (11.1%); and >15 years, 25 (2.6%). A total of 509 (52.3%) participants were male, and 464 (47.6%) were female. A total of 528 (54.2%) participants were outpatients, while 446 (45.8%) were inpatients.

### 3.2 Viral identification

**FIGURE 1:**
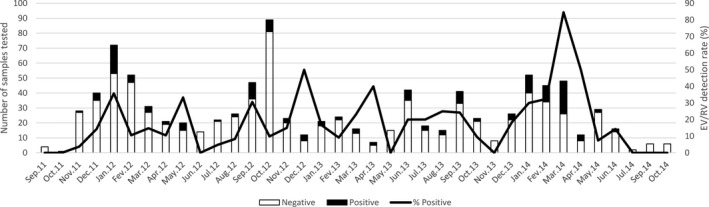
Seasonal distribution of Rhinoviruses and/or Enteroviruses detected in patients with acute respiratory infections during September 2011 till October 2014 in Cameroon. Primary *y*‐axis and bars describe the number of cases, while secondary *y*‐axis and lines describe the monthly detection rate

Of the 974 swabs tested, 160 (16.4%) were positive for RVs and/or EVs (Table 1). The rate of RV/EV positivity in inpatients vs outpatient was significantly different (*P* = .015). The rate of RV/EV positivity with respect to the age (*P* = .023) and year of recruitment was also significantly different (*P* = .002). In contrast, no significant difference in RV/EV positivity was found with respect to age group (*P* = .291) or sex (*P* = .604). The most common symptoms recorded in participants were rhinorrhoea and cough. RVs and EVs were found to circulate throughout the year (Figure 1). Apart from fatigue (*P* = .030) and headaches (*P* = .004), all other symptoms recorded were not significantly associated with RV/EV infections.

**TABLE 1:** General data of the study participants

Characteristics	Total	Rhinovirus/Enterovirus		*P*‐value
		Negative	Positive	
	No (974)	814 (83.6)	160 (16.4)	
Gender				
Male	509 (52.3)	422 (51.8)	87 (54.4)	.604
Female	464 (47.6)	391 (48.0)	73 (45.6)	
Age				
Median [IQR]	1.7 [0.7‐3.0]	2.0 [0.8‐4.0]	1.4 [0.6‐3.0]	.023
Age				
0‐2 y	597 (61.3)	492 (60.4)	105 (65.6)	.290
2‐5 y	238 (24.4)	200 (24.6)	38 (23.8)	
5‐15 y	108 (11.1)	93 (11.4)	15 (9.4)	
>15 y	25 (2.6)	24 (2.9)	1 (0.6)	
NA	6 (0.6)	5 (0.6)	1 (0.6)	
Patient				
Inpatients	446 (45.8)	387 (47.5)	59 (36.9)	.015
Outpatients	528 (54.2)	427 (52.5)	101 (63.1)	
Year				
2011	73 (7.5)	67 (8.2)	6 (3.8)	.002
2012	429 (44.0)	365 (44.8)	64 (40.0)	
2013	256 (26.3)	219 (26.9)	37 (23.1)	
2014	216 (22.2)	163 (20.0)	53 (33.1)	
Symptoms				
Rhinorrhoea	788 (80.9)	650 (79.9)	138 (86.3)	.199
Cough	819 (84.1)	685 (84.2)	134 (83.8)	.999
Fatigue	553 (56.8)	474 (58.2)	79 (49.4)	.030
Wheezing	369 (37.9)	305 (37.5)	64 (40.0)	1.000
Vomiting	288 (29.6)	244 (30.0)	44 (27.5)	.318
Breathlessness	205 (21.0)	170 (20.9)	35 (21.9)	.935
Diarrhoea	191 (19.6)	157 (19.3)	34 (21.3)	1.000
Sore throat	177 (18.2)	156 (19.2)	21 (13.1)	.100
Cutaneous rash	82 (8.4)	63 (7.7)	19 (11.9)	.200
Headache	138 (14.2)	126 (15.5)	12 (7.5)	.004
Conjunctivitis	102 (10.5)	90 (11.1)	12 (7.5)	.134
Earache	46 (4.7)	37 (4.5)	9 (5.6)	.501
Arthralgia	63 (6.5)	57 (7.0)	6 (3.8)	.184
Myalgia	39 (4.0)	36 (4.4)	3 (1.9)	.148

Note

Data are number (%).

Abbreviation: NA, Not available.

### 3.3 Genotyping

Overall, 43 RV/EV positive samples were available for molecular characterization in this study. The 5′UTR‐VP4‐VP2 region was amplified and sequenced successfully for 27 (62.8%) samples (22 RV and five EV) out of the 43 tested. The remaining 16 RV/EV samples from which these target viruses could not be sequenced exhibited a relatively high cycles threshold ranging from 31.5 to 37.5 in RV/EV real‐time PCR detection. Of these 16 samples that could not be successfully sequenced, three could not be amplified by conventional RT‐PCR and 13 produced inexploitable electrophoregrams with superimposed peaks.

**FIGURE 2:**
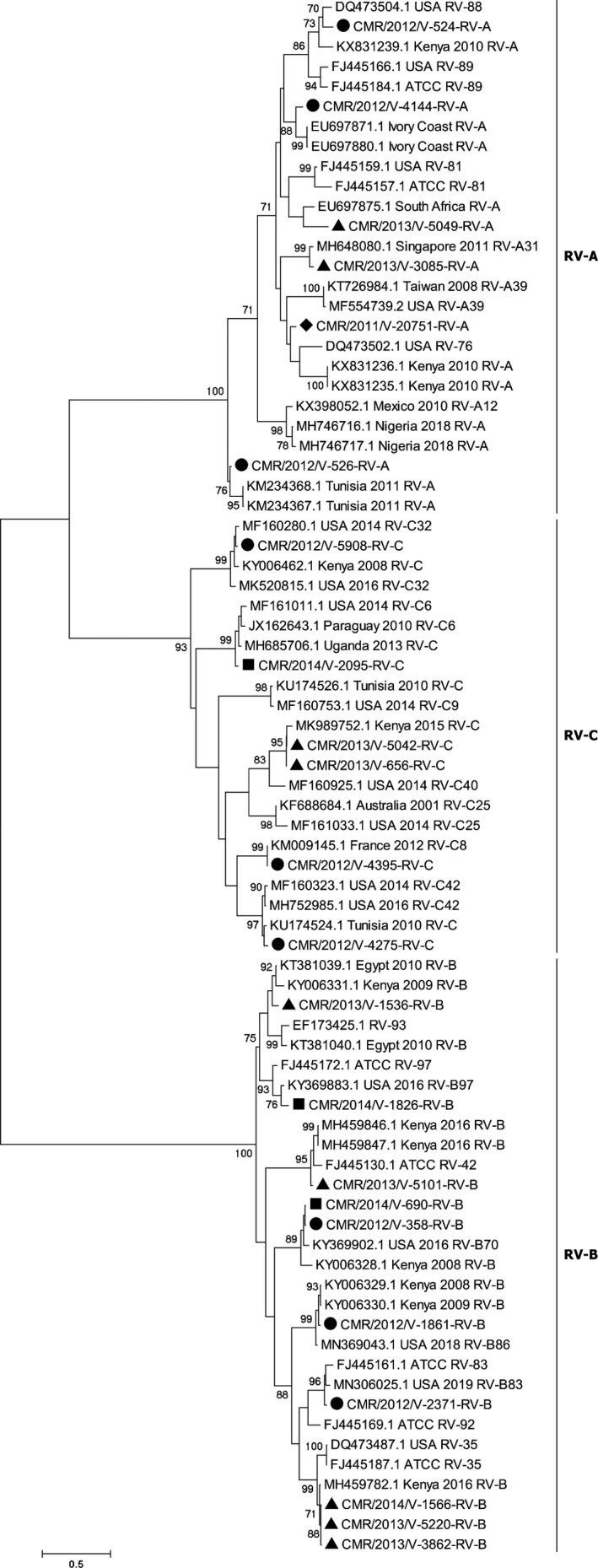
Phylogenetic tree of Rhinoviruses strains detected in Yaoundé, Cameroon, from 2011 to 2014. Multiple sequence alignment was performed with Clustal W. The rooted tree was generated based on VP4/VP2 gene nucleotide sequences using the maximum likelihood method and the best model evolution (General Time Reversible) with a Gamma distribution and assuming that a certain fraction of sites was invariable on an evolutionary level in Mega 6. The scale bars represent the frequency of nucleotide substitutions, and the numbers on the nodes of the branches are determined values for resampling bootstrap after 1000 iterations. Only values ≥70% are presented. The reference sequences of different continents, obtained on GenBank, are identified from the left to the right with the access number, the origin country and the species attributed by the author. The strains of the current study are designated from left to right by CMR (Cameroon), the year of detection and the laboratory number. Cameroon sequences are shown with (♦), (●), (▲) and (■) for the 2011, 2012, 2013 and 2014 sequences, respectively

Rhinovirus‐A, B and C species circulated in Cameroon during the 3 years of the study. This study identified six RV‐A, 10 RV‐B and six RV‐C (Table 2). The six RV types of the species RV‐A comprised A31, A76, A88, A81, along with two unassigned types. One of these unassigned RV‐A types was strikingly related to their closest matches from Tunisia, whereas the other one was featured closest phylogenetic relatedness with isolates from Ivory Coast (Figure 1). The 10 studied RVs of the RV‐B species comprised 3 B35, B42, B70, B83, B86, B93, B97 and one unassigned. The six virus types of the RV‐C species included RV‐C6, C8, C32, C40 and C42. All studied RV isolates segregated into diverse phylogenetic groups defined by their homotypic reference isolates (Figure 2). A nucleotide diversity of 2.9%‐19.1%, 0.3%‐35.0% and 0.0%‐41.0% was featured between the studied sequences and their homotypic reference sequences of the RV‐A, RV‐B and RV‐C species, respectively. Studied sequences and other reference sequences of the RV‐A, RV‐B and RV‐C displayed an amino acid diversity of 0.0%‐10.8%, 0.0%‐17.3% and 0.0%‐21.3%, respectively.

**TABLE 2:** Characteristic of RV/EV successfully sequenced samples

Characteristics	EV (5)	RV‐A (6)	RV‐B (10)	RV‐C (6)	Total
Types	Coxsackievirus‐B1 (2), Coxsackievirus‐A9 (1), Coxsackievirus‐A5 (1) and EV‐C117 (1)	A31, A76, A88, A81 and 2A non‐typed	3 B35, B42, B70, B83, B86, B93 and B97	C6, C8, C32, C40, and C42	
Gender					
Male	3	6	3	4	16
Female	2	0	7	2	11
Age					
0‐2 y	2	4	6	5	17
2‐5 y	2	1	2	1	6
5‐15 y	1	1	2	0	4
Patient					
Inpatients	0	3	3	4	10
Outpatients	5	3	7	2	17
Year					
2011	0	1	0	0	1
2012	3	3	3	3	12
2013	0	2	4	2	8
2014	2	0	3	1	6

**FIGURE 3:**
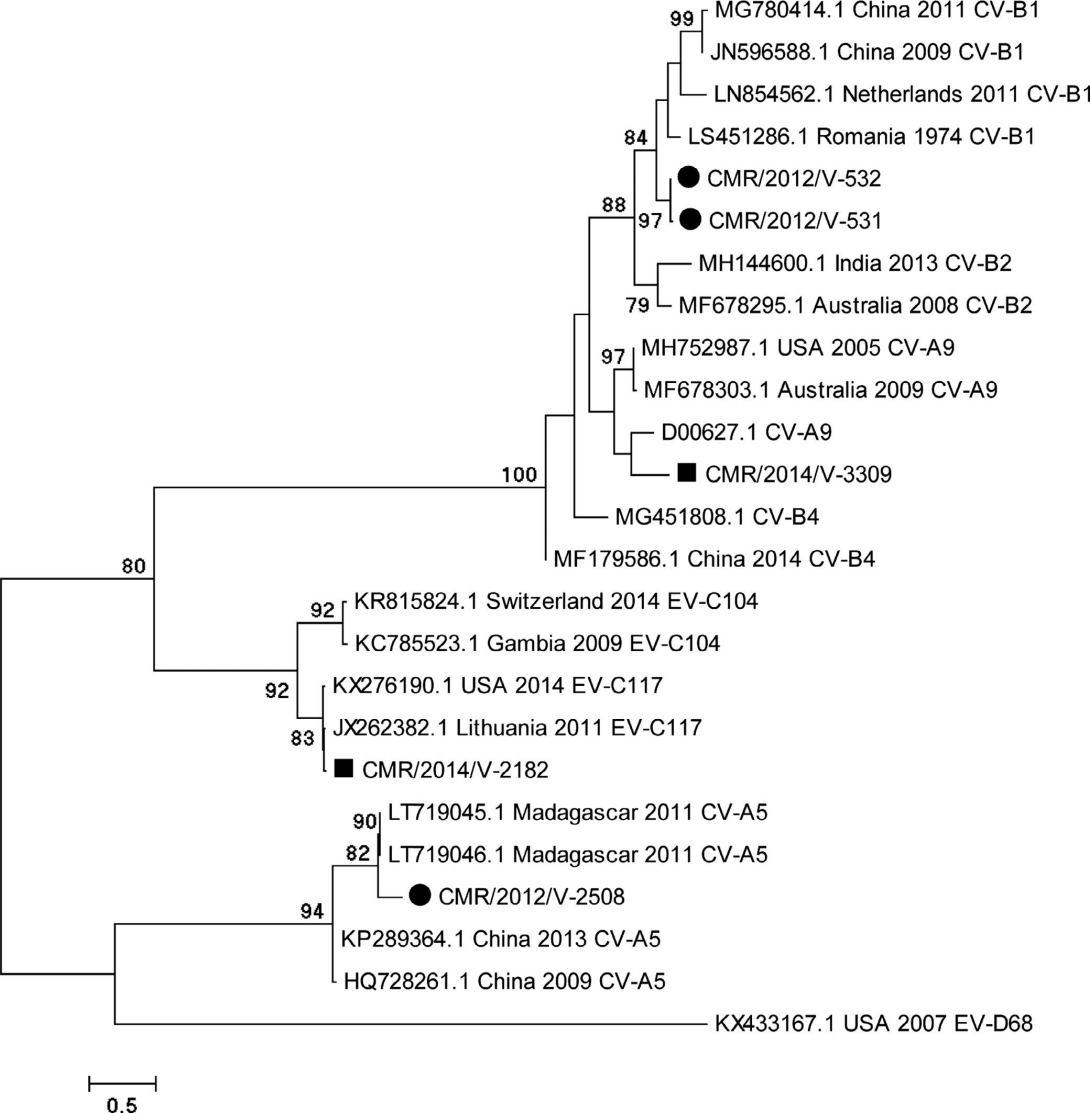
Phylogenetic tree of Enteroviruses strains detected in Yaoundé, Cameroon, from 2011 to 2014. Multiple sequence alignment was performed with Clustal W. The rooted tree was generated based on VP4/VP2 gene nucleotide sequences using the maximum likelihood method and the best model evolution (General Time Reversible) with a Gamma distribution and assuming that a certain fraction of sites was invariable on an evolutionary level in Mega 6. The scale bars represent the frequency of nucleotide substitutions, and the numbers on the nodes of the branches are determined values for resampling bootstrap after 1000 iterations. Only values ≥70% are presented. The reference sequences of different continents, obtained on GenBank, are identified from the left to the right with the access number, the origin country and the species attributed by the author. The strains of the current study are designated from left to right by CMR (Cameroon), the year of detection and the laboratory number. Cameroon sequences are shown with (●) and (■) for the 2012 and 2014 sequences, respectively

The five successfully sequenced EV isolates were from outpatients. The phylogenetic tree showed that the five EVs belonged to the Coxsackievirus‐B1 (2), Coxsackievirus‐A9 (1), Coxsackievirus‐A5 (1) and EV‐C117 (1) types (Figure 2). A nucleotide sequence similarity of 1.4%‐65.7% was recorded between the studied and reference EV sequences. As expected, all studied EV isolates segregated into diverse phylogenetic groups defined by their homotypic reference isolates (Figure 3).

## 4 DISCUSSION

This study reports for the first time in Cameroon the molecular characterization of RV and EV strains detected in hospitalized and ambulatory patients of all ages with acute respiratory infections. A total of 16.4% of patients tested in this study presented with RV and/or EV infections. Data analysis showed a high prevalence of RV/EV in outpatients and a year‐round circulation of RV/EV strains in Cameroon. The strains of RV and EV successfully sequenced suggested a high diversity of RV and EV types circulating in Cameroon.

The prevalence of these two viruses varied with respect to multiple factors including, patient age, clinical presentation, type of sampling, climate, time of year, type of detection assay, species/types/genotypes of the studied viruses, the type of sample tested and many other factors.^27^, ^49^, ^50^ As expected from previous studies, RV and EV detection rates were lower in adults compared to children likely because adult have experienced multiple past exposure to these widespread viruses and are thus less susceptible to infections. Accordingly, Aston et al^49^ in a study including adults >18 years hospitalized with pneumonia in Malawi reported a detection frequency as low as 3.7% (17/455) for RV and 1.1% (5/455) for EV by PCR using nasopharyngeal swabs. As in this study a high RV/EV detection rate at 25.3% (41/162) was also found for EV in bronchial, throat and/or nasal specimens from children <5 years with acute respiratory infections in Senegal.^50^ Like in most regions of the world including Africa, RV/EV circulated throughout the year during this study.^51^, ^52^ However, EVs in temperate countries show peaks in summer and fall, while in tropical countries peaks are observed in the rainy season.^52^ RVs also exhibit the same annual circulation pattern but with peaks recorded in autumn and spring.^53^, ^54^ The symptoms (fatigue and headaches) found associated with RV/EV positivity in this study are general signs corresponding to several other diseases. This suggests that it is difficult to derive a fair interpretation. However, none of the respiratory infections specific symptoms were associated with RV/EV positivity. This is because all of the patients had acute respiratory infections, and the RV/EV negatives are thus positive for other respiratory pathogens that cause similar signs. Successful sequencing rates of RV and/or EV positive samples ranging from 23.6% to 100% can be obtained in studies.^51^, ^52^, ^55^, ^56^, ^57^ Brini Khalifa et al^27^ in a study including children <1 year with acute respiratory infections in Tunisia reported a RV detection rate in nasopharyngeal swabs as high as 51.8% (267/515) by PCR, thus indicating that your age is a key factor associated to RV and EV infection. This large variability in the RV and/or EV detection and sequencing rates in studies depends on several factors including mainly the detection assay used, viral load, type of samples tested and target genes. Altogether, this and previous studies indicate that the occurrence of RV and EV is more frequent high in children compared to adults.

In this study, only 62.8% (27/43) of the RV and EV detected by real‐time PCR were successfully sequenced. Samples that could not amplify in conventional PCR were those with low viral loads indicated by high cycles thresholds ranging from 31.5 to 37.5. This is consistent with the fact that real‐time RT‐PCR is more sensitive than conventional RT‐PCR.

The pattern of RV/EV circulation throughout the year observed in this study is very common in most studies.^51^, ^52^, ^57^ Regardless of the type of sample considered and the sequencing rate of RV strains detected in most studies in Africa, RV‐A and RV‐C strains are generally predominant compared to RV‐B species.^30^, ^51^, ^52^, ^55^, ^56^, ^57^ However, RV‐B strains were predominant in this study. However, this result should be considered with caution since there is a potential bias associated to the lack of sufficient sample volume for most RV and EV detected and the low viral loads preventing successful sequencing of some samples. The EV types identified in this study have already been described in other studies recruiting patients with acute respiratory infections.^39^, ^58^, ^59^, ^60^, ^61^, ^62^ In particular, the EV‐C117 which was first described in 2012 in a child with pneumonia^60^ was found identified in a ILI case in this study. Apart from CV‐B1, other EV type identified in ARI patients in this study has not been reported in previous studies among healthy and acute flaccid paralysis patients in Cameroon.^63^, ^64^, ^65^ It is noteworthy that we identify no EV‐D68 isolate despite the epidemiological context of the emergence EV‐68 in patients with acute respiratory infections in 2014.^16^

The small number of typed RV/EV positive samples and the potential bias in the samples available for molecular typing represent the main limitations of this study. Although apparently unrealistic, systematic typing of RV and EV strains in Cameroon would have likely revealed a more comprehensive genetic landscape of the RV and EV diversity in Cameroon. Nevertheless, the findings of this study suggest that RV and EV are common in Cameroon and they feature a high genetic variability in term of co‐circulating RV and EV types and species. Larger studies are still needed to further explore the molecular epidemiology of RV and EV in ARI patients in Cameroon using extended sample collections and more powerful sequencing approaches such as next generation sequencing.

In conclusion, the findings of the present study suggest an important level of annual circulation of respiratory RV/EV in Cameroon. Molecular characterizations have revealed a large diversity of RV/EV strains.

## CONFLICT OF INTEREST

None.

## AUTHOR CONTRIBUTION

**Sebastien Kenmoe:** Data curation (lead); Formal analysis (lead); Investigation (lead); Methodology (lead); Project administration (lead); Validation (lead); Writing‐original draft (lead); Writing‐review & editing (lead). **Serge Alain Sadeuh‐Mba:** Methodology (supporting); Validation (supporting); Writing‐review & editing (supporting). **Marie‐Astrid Vernet:** Methodology (lead); Validation (supporting); Writing‐review & editing (supporting). **Véronique Penlap Beng:** Methodology (supporting); Validation (supporting); Writing‐review & editing (supporting). **Astrid Vabret:** Methodology (lead); Project administration (lead); Supervision (lead); Validation (supporting); Writing‐review & editing (supporting). **Richard Njouom:** Conceptualization (lead); Methodology (supporting); Project administration (lead); Supervision (lead); Writing‐review & editing (supporting).

### PEER REVIEW

The peer review history for this article is available at https://publons.com/publon/10.1111/irv.12851.

## Supporting information

Figure S1

Figure S2

## Data Availability

The data that supports the findings of this study are available in the supplementary material of this article.
