# Supplementary material for: Molecular epidemiology of Enteroviruses and Rhinoviruses in patients with acute respiratory infections in Yaounde, Cameroon
Source: Influenza Other Respir Viruses. 2021 Mar 10;15(5):641–50. doi: 10.1111/irv.12851 (PMC8404047; doi:10.1111/irv.12851)
Supplement: Supplementary file 1 [file IRV-15-641-s001.doc]

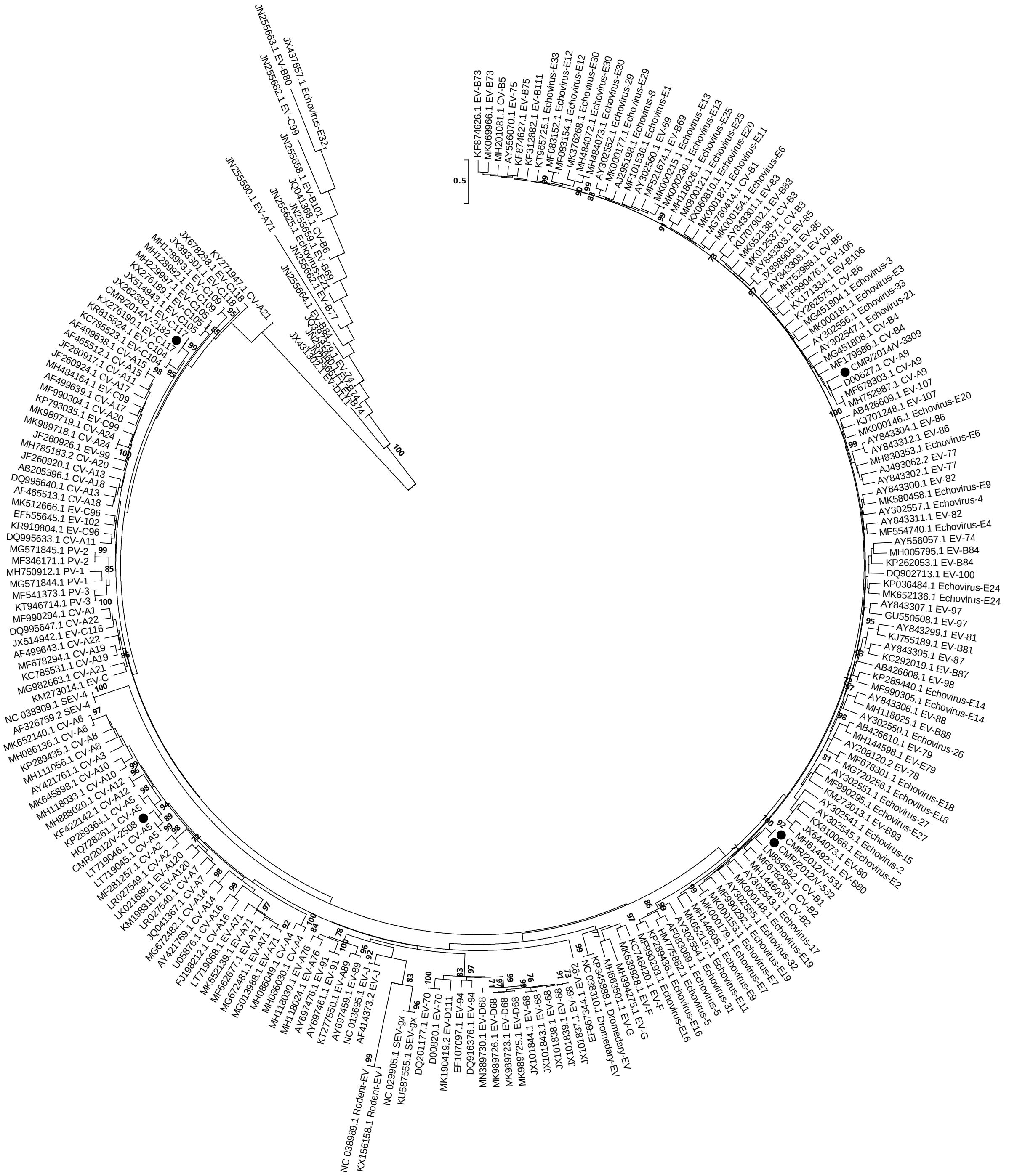
0.5
KF874626.1 EV-B73
MK069966.1 EV-B73
MH201081.1 CV-B5
AY556070.1 EV-75
KF874627.1 EV-B75
KF312882.1 EV-B111
CMR/2014/V-3309
D00627.1 CV-A9
MF678303.1 CV-A9
MH752987.1 EV-107
AB426609.1 EV-107
KJ701248.1 Echovirus-E20
MK000146.1 EV-86
AY843304.1 EV-86
AY843312.1 Echovirus-E6
MH830353.1 Echovirus-E6
AJ493062.2 EV-77
AY843302.1 EV-77
AY843300.1 EV-82
MK580458.1 Echovirus-E9
AY302557.1 Echovirus-4
AY843311.1 EV-82
MF554740.1 Echovirus-E4
AY556057.1 EV-74
MH005795.1 EV-B84
KP262053.1 EV-B84
DQ902713.1 EV-100
KP036484.1 Echovirus-E24
MK652136.1 Echovirus-E24
AY843307.1 EV-97
GU550508.1 EV-97
AY843299.1 EV-81
MG571845.1 PV-2
MF346171.1 PV-2
MH750912.1 PV-1
MG571844.1 PV-1
MF541373.1 PV-3
KT946714.1 PV-3
MF990294.1 CV-A1
NC 038989.1 Rodent-EV
KX156158.1 Rodent-EV
NC 029905.1 SEV-gx
KU587555.1 SEV-gx
JN255590.1 EV-A71
JX437657.1 Echovirus-E32
