# Supplementary material for: Molecular epidemiology of Enteroviruses and Rhinoviruses in patients with acute respiratory infections in Yaounde, Cameroon
Source: Influenza Other Respir Viruses. 2021 Mar 10;15(5):641–50. doi: 10.1111/irv.12851 (PMC8404047; doi:10.1111/irv.12851)
Supplement: Supplementary file 2 [file IRV-15-641-s002.doc]

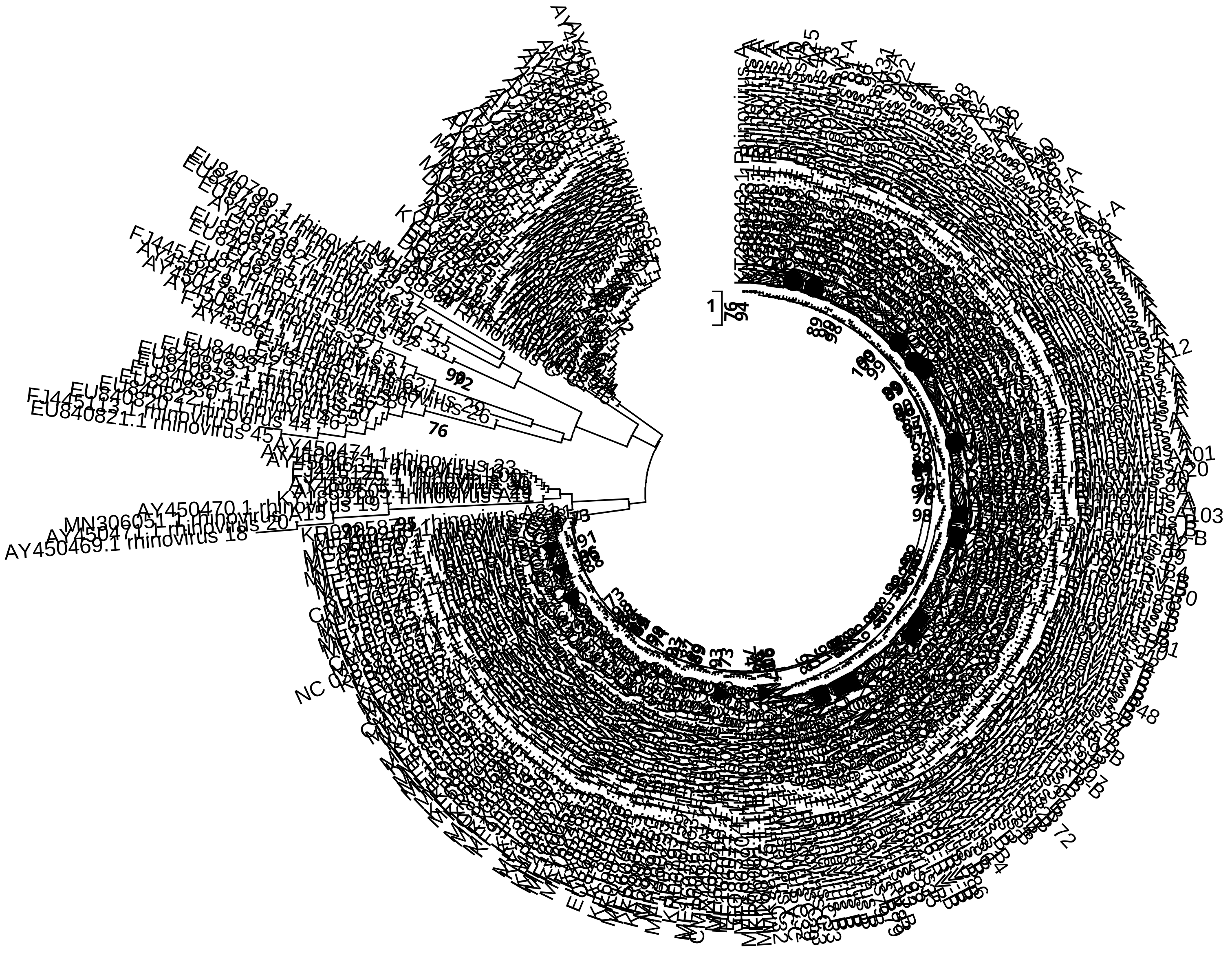

AY450469.1 rhinovirus 18
